# Mixed reality improves agreement on surgical approach selection and patient positioning in tibial plateau fracture planning compared to CT, 3DCT and 3D printing

**DOI:** 10.1007/s00068-026-03230-4

**Published:** 2026-06-22

**Authors:** Tobias Dust, Julian-Elias Henneberg, Maximilian Hartel, Alonja Reiter, Julian Kylies, Alexander Korthaus, Anna Streckenbach, Johannes Keller, Karl-Heinz Frosch, Matthias Krause

**Affiliations:** 1 Department of Trauma and Orthopaedic Surgery, University Medical Center Hamburg – Eppendorf, Hamburg, Germany; 2 Department of Trauma Surgery, Orthopaedics and Sports Traumatology, BG Hospital Hamburg, Hamburg, Germany; 3 Department of Diagnostic and Interventional Radiology and Nuclear Medicine, University Medical Center Hamburg-Eppendorf, Hamburg, Germany

**Keywords:** Tibial plateau fracture, Mixed reality, Preoperative planning, Patient positioning, Surgical approach, Interobserver agreement

## Abstract

**Purpose:**

Tibial plateau fractures require precise preoperative planning, particularly regarding treatment concept, patient positioning, and surgical approach. Mixed reality (MR) may enhance three-dimensional understanding of fracture morphology beyond conventional imaging and physical 3D models. This study investigated whether MR improves agreement on key preoperative planning decisions compared to computed tomography (CT), 3D computed tomography (3DCT), and 3D-printed fracture models.

**Methods:**

Twelve orthopaedic trauma surgeons (6 junior, 6 senior) evaluated 22 surgically treated tibial plateau fractures (AO/OTA type B or C). Each case was assessed in four steps using (1) CT, (2) 3DCT reconstructions, (3) physical 3D-printed models, and (4) MR visualization using Microsoft Hololens 2. Raters selected (i) treatment concept, (ii) patient positioning, and (iii) surgical approach. Interobserver agreement was analysed using Fleiss’ kappa (κ) and percentage match (PM).

**Results:**

Mixed Reality (MR) yielded the highest interobserver agreement for both surgical approach (PM 32%, κ = 0.30) and patient positioning (PM 57%, κ = 0.35), particularly among junior surgeons (approach PM 39%, κ = 0.28; positioning PM 57%, κ = 0.39). Compared to CT, 3DCT, and 3D printing, MR demonstrated consistent improvements, while agreement on treatment concepts remained high across all modalities (PM > 82%).

**Conclusion:**

Mixed Reality (MR) improved interobserver agreement in preoperative planning for tibial plateau fractures, particularly for surgical approach selection and patient positioning. These effects were most pronounced among less experienced raters, highlighting MR’s role as a cognitive support tool and a promising instrument in surgical education. Compared to conventional imaging and 3D printing, MR provided dynamic interaction and greater planning flexibility. While further clinical validation is required, these findings support MR as a valuable adjunct in orthopaedic trauma planning.

**Level of evidence:**

Level II.

**Supplementary Information:**

The online version contains supplementary material available at 10.1007/s00068-026-03230-4.

## Introduction

The surgical treatment of tibial plateau fractures remains one of the most demanding tasks in orthopaedic trauma surgery [1]. Due to the complex three-dimensional fracture morphology and frequent involvement of posterior and posterolateral segments, accurate preoperative planning is essential to achieve anatomical reduction, stable fixation, and appropriate functional outcomes [2–5]. In particular, the choice of treatment concept, patient positioning, and surgical approach plays a decisive role in enabling adequate exposure of the fracture site and avoiding intraoperative compromises that may negatively affect reduction quality and implant placement [6–9].

Computed tomography (CT) and three-dimensional computed tomography (3DCT) reconstructions have become standard tools for the assessment of tibial plateau fractures and substantially improved fracture visualization compared with conventional radiography [10]. Nevertheless, important aspects of spatial fracture understanding and fragment interrelation may remain difficult to appreciate using two-dimensional screens, especially in complex fracture patterns. To overcome these limitations, point-of-care 3D printing has been introduced as an additional technology for preoperative planning [11–14]. Previous studies have demonstrated that physical 3D-printed fracture models can enhance the surgeon’s spatial understanding and may improve agreement regarding patient positioning and surgical approach selection, particularly among less experienced surgeons [11, 12, 15]. However, 3D printing is associated with additional costs, production time, and logistical constraints, which may limit its routine clinical use [13, 16].

More recently, immersive visualization technologies such as Virtual Reality (VR) and Mixed Reality (MR) have gained increasing attention in orthopaedic trauma surgery [17–20]. These technologies allow interactive 3D visualization of patient-specific fracture anatomy without the need for physical model production. While VR and MR have already been investigated for fracture diagnostics and classification tasks [18, 19], evidence regarding their role in preoperative decision making remains limited. In particular, it is currently unclear whether MR can provide a comparable or even superior benefit to 3D printing when planning critical operative parameters such as treatment concept, patient positioning, and surgical approach.

Therefore, the purpose of this study was to assess the impact of Mixed Reality on preoperative planning of tibial plateau fractures in comparison with CT, 3DCT, and physical 3D-printed models. Specifically, we aimed to analyze interobserver agreement regarding (1) treatment concept, (2) patient positioning, and (3) surgical approach selection using these different visualization modalities. We hypothesized that Mixed Reality would improve agreement on key preoperative planning decisions compared with conventional imaging techniques and physical 3D-printed models, with potential differences across varying levels of surgical experience.

## Methods

### Study design

This study was designed as a retrospective observer study evaluating the impact of different visualisation modalities on preoperative planning of tibial plateau fractures. Conventional CT, 3DCT, physical 3D-printed fracture models, and MR visualisation were compared with regard to agreement on key preoperative planning decisions.

### Fracture cases

A total of 22 intra-articular tibial plateau fractures (AO/OTA type B or C) treated between 2017 and 2022 were included. Eligibility required availability of a preoperative CT scan with an axial slice thickness of 1 mm or less and complete multiplanar reconstructions. Cases with previous fractures of the proximal tibia or incomplete imaging data were excluded. All datasets were fully anonymised prior to analysis.

### Imaging data and three-dimensional reconstructions

CT datasets were obtained from the institutional picture archiving and communication system (PACS) and stored in Digital Imaging and Communications in Medicine (DICOM) format. Raters reviewed axial, sagittal and coronal CT image stacks in a scrollable format simulating standard PACS navigation.

3DCT reconstructions of the proximal tibia and fibula were generated from the same datasets. The femur and patella were digitally removed to improve visualisation of the tibial plateau and fracture morphology. The 3D reconstructions were presented as rotatable models allowing free inspection from different perspectives.

### Segmentation and 3D printing

Fracture segmentation was performed using Materialise Mimics Innovation Suite (version 24; Materialise, Leuven, Belgium) (Supplement 1). A threshold-based semi-automatic segmentation approach was applied to isolate osseous structures, followed by manual refinement in cases of comminution or depressed fragments. Post-processing was carried out using Materialise 3-matic Medical (version 16; Materialise, Leuven, Belgium) (Supplement 2, A & B), including surface smoothing and stabilisation of fracture fragments where required.

The segmented fracture models were exported as standard tessellation language (STL) files and printed at a 1:1 scale using an Ultimaker S5 dual-head fused deposition modelling (FDM) printer (Ultimaker, Utrecht, The Netherlands) (Supplement 2, C & D). Polylactic acid (PLA) was used as the primary printing material, while water-soluble polyvinyl alcohol (PVA) served as support material. A layer thickness of 0.1 mm was selected to ensure high geometric accuracy and sufficient detail of the fracture morphology. After printing, support structures were removed prior to evaluation. The physical 3D-printed models were used exclusively for visual and tactile inspection during the respective study phase. No digital images or photographs of the printed models were provided to the raters.

### Mixed reality visualisation

Mixed reality visualisation was performed using the Microsoft HoloLens 2 head-mounted display. Patient-specific 3D fracture models generated from the segmentation workflow were imported into the Materialise Viewer application (Supplement 3). Raters were able to interact with the virtual fracture models using standard MR gestures, including rotation, scaling, and free spatial positioning within the real-world environment. All raters received a standardised introduction to the MR system prior to the study.

### Raters

Twelve orthopaedic trauma surgeons participated in the study, including six junior and six senior surgeons. All raters were blinded to the actual surgical treatment performed in the clinical cases.

### Study procedure

Each rater evaluated all 22 fracture cases sequentially using four different visualisation modalities:

1. CT,
2. 3DCT,
3. physical 3D-printed model, and
4. Mixed Reality visualisation.

The order of the modalities was identical for all raters. At each stage, raters were asked to independently determine three preoperative planning parameters:

- Treatment concept,
- Patient positioning, and.
- Surgical approach.

Predefined answer options were provided for each parameter to ensure standardised assessment across raters using an online questionnaire (Supplement 4). Multiple predefined surgical approaches could be selected if a combined approach was considered necessary. Free-text responses were not permitted for treatment or planning decisions in order to maximize reproducibility and comparability between raters. However, optional comment fields were available for technical feedback related to questionnaire functionality or image display issues.

The questionnaire was developed internally by an expert panel and had previously been used in earlier studies investigating 3D visualization techniques in tibial plateau fractures [11, 12, 19]. No formal pilot testing of the questionnaire was performed prior to study initiation. Raters were not permitted to revise previous answers after progressing to the next stage.

#### Outcome measures

The primary outcome measure was interobserver agreement for surgical approach selection. Secondary outcome measures included interobserver agreement for patient positioning and treatment concept selection.

#### Statistical analysis

Interobserver agreement was analysed using Fleiss’ kappa (κ) for each outcome parameter and visualisation modality. Kappa values were interpreted according to the criteria proposed by Landis and Koch (Table 1). In addition, percentage match (PM) was calculated as a descriptive measure of agreement, representing the proportion of identical ratings among raters.

**Table 1:** Landis and Koch grading of reliability based on κ co-efficient values [39]

κ co-efficient	Reliability grading
< 0.00	poor
0.01–0.20	slight
0.21–0.40	fair
0.41–0.60	moderate
0.61–0.80	substantial
> 0.80	excellent

Agreement analyses were performed for the entire rater cohort as well as separately for junior and senior surgeons. Statistical analyses were conducted using SPSS Statistics (version 26; IBM Corp., Armonk, NY, USA). No formal sample size calculation was performed. The number of included cases was determined pragmatically based on the availability of complete imaging datasets and the feasibility of segmentation, 3D printing, and MR model preparation across all modalities.

### Ethics approval

The study was approved by the Ethics Committee of the Medical Chamber of Hamburg, Germany (ID: 2024-101279-BO-ff) and was conducted in accordance with the Declaration of Helsinki. All imaging data were anonymised prior to analysis.

## Results

### Interobserver agreement for surgical approach selection

Interobserver agreement for surgical approach selection, defined as the primary outcome parameter, is summarised in Table 2. Overall agreement was low to moderate across all visualisation modalities. Percentage match (PM) was 29% for both CT and 3DCT, increased to 33% for 3D-printed models, and reached 32% for MR. Fleiss’ kappa values showed a comparable pattern, ranging from 0.22 for 3DCT to 0.30 for MR.

**Table 2:** Interobserver agreements for the surgical approach

Surgical approach	CT		3DCT		3D		MR	
	PM	κ	PM	κ	PM	κ	PM	κ
Overall	29%	0.23	29%	0.22	33%	0.27	32%	0.30
Junior Surgeons	27%	0.19	27%	0.19	34%	0.26	39%	0.28
Senior Surgeons	33%	0.27	33%	0.26	32%	0.26	31%	0.21

When stratified by level of experience, junior surgeons demonstrated lower agreement across all modalities. In this subgroup, PM increased from 27% with CT and 3DCT to 34% with 3D-printed models and to 39% with MR, while kappa values increased from 0.19 to 0.28. Among senior surgeons, agreement remained relatively stable across modalities, with PM values between 31% and 33% and kappa values between 0.21 and 0.27.

### Interobserver agreement for patient positioning

Interobserver agreement for patient positioning is presented in Table 3. Across all raters, PM was 46% for both CT and 3DCT, increased to 53% with 3D-printed models, and further increased to 57% with MR. Corresponding kappa values were 0.25 for CT, 0.26 for 3DCT, 0.36 for 3D printing, and 0.35 for MR.

**Table 3:** Interobserver agreements for the patient positioning

Patient positioning	CT		3DCT		3D		MR	
	PM	κ	PM	κ	PM	κ	PM	κ
Overall	46%	0.25	46%	0.26	53%	0.36	57%	0.35
Junior Surgeons	42%	0.19	43%	0.23	52%	0.35	57%	0.39
Senior Surgeons	55%	0.36	53%	0.33	55%	0.36	55%	0.27

Junior surgeons demonstrated a marked increase in agreement with advanced visualisation modalities. In this subgroup, PM increased from 42% with CT to 52% with 3D printing and to 57% with MR, while kappa values increased from 0.19 to 0.39. In contrast, senior surgeons showed higher baseline agreement, with PM values ranging from 53% to 55% and kappa values between 0.27 and 0.36, without substantial differences between modalities.

### Interobserver agreement for treatment concept

Interobserver agreement for treatment concept selection is shown in Table 4. Agreement was high across all visualisation modalities. Overall percentage match (PM) values ranged from 82% to 91%, with the highest PM observed for MR. Fleiss’ kappa values were low to moderate and ranged from 0.17 to 0.25 across modalities.

**Table 4:** Interobserver agreements for the treatment concept

Treatment concept	CT		3DCT		3D		MR	
	PM	κ	PM	κ	PM	κ	PM	κ
Overall	83%	0.23	83%	0.25	82%	0.17	91%	0.11
Junior Surgeons	82%	0.20	82%	0.21	81%	0.16	91%	-0.3
Senior Surgeons	83%	0.24	84%	0.25	83%	0.15	92%	0.23

Similar patterns were observed in both junior and senior surgeons. Junior surgeons demonstrated PM values between 81% and 91%, whereas senior surgeons showed PM values between 83% and 92%. Kappa values remained within a comparable range across all modalities and experience levels.

## Discussion

The primary finding of this study is that Mixed Reality (MR) improved interobserver agreement in preoperative planning for tibial plateau fractures, particularly in the domains of surgical approach selection and patient positioning. While agreement on treatment concepts was already high across all modalities MR showed the clearest improvements in surgical approach selection and patient positioning—two areas where interobserver agreement was lowest with conventional modalities. These benefits were most pronounced among less experienced raters, suggesting that MR offers a valuable cognitive support tool for surgical planning. Although we observed consistent trends favoring MR, the study was not powered to assess statistical significance. In this context, the observed advantages of MR may not solely reflect modality-specific effects, but also the cumulative benefit of sequential multimodal fracture assessment, with MR representing the final and most information-rich visualization step within the assessment workflow.

Correct patient positioning and surgical access are foundational to optimal outcomes in intra-articular fracture management. Mispositioning can compromise intraoperative visualization, increase operative time, and complicate access to key fracture zones [13, 15, 21–24]. In tibial plateau fractures—especially those involving posterior fragments—optimal positioning (e.g., prone vs. supine, flexion angle) determines whether an anatomic reduction can be achieved without excessive soft tissue dissection [25].

Likewise, choosing the correct surgical approach is critical for preserving soft tissue integrity, minimizing neurovascular injury, and enabling stable implant positioning [9, 21, 23, 26]. Manidakis et al. further linked suboptimal treatment strategies to poor functional recovery and persistent postoperative pain [27]. Our findings support that MR, by enhancing spatial understanding, may reduce these risks through more accurate preoperative access planning. Although not measured directly in our study, this planning accuracy has the potential to translate into improved intraoperative efficiency and patient safety.

Our findings align with and expand upon earlier work on 3D visualization in orthopaedic trauma care. Prior studies have shown that 3D printing can improve diagnostic confidence, spatial understanding, and decision-making in managing intra-articular fractures [12, 13, 15, 19]. For instance, 3D printing improved rater agreement in surgical approach and patient positioning decisions, especially among less experienced users [12, 15]. Additional research on acetabular and pilon fractures supports similar trends [28–30].

MR appears to extend these benefits. In contrast to the static inspection of physical models, MR offers interactive and dynamic fracture exploration, allowing users to manipulate holograms within the surgical field of view. Prior evidence for the preoperative utility of MR in orthopaedics remains limited. Bitschi et al. evaluated MR in tibial plateau fractures and found that visualization with MR changed the fracture segment classification in 79% of cases using the 10-segment system, suggesting that dynamic holographic exploration may impact decision-making more than conventional modalities [18].

In contrast, Brouwers et al. conducted a study on acetabular fractures comparing VR models to 3D printing [17]. Their findings revealed that VR did not surpass 3D printing in terms of planning accuracy or user confidence, highlighting limitations in the standardization and usability of immersive technologies. These contrasting results underscore the need to interpret MR outcomes in the context of hardware and software variation. While Brouwers et al. did not find a clear advantage of immersive environments over 3D printing in terms of planning accuracy, our results suggest that MR may offer superior value in dynamic planning contexts. Additionally, earlier work has demonstrated improved diagnostic accuracy and user confidence with MR in fracture classification, which further supports our findings in the preoperative planning domain [19].

Consistent with previous work, our results highlight that MR’s benefits are most pronounced among junior surgeons. This aligns with prior studies who found that 3D technologies enhanced spatial comprehension and improved decision-making in less experienced surgeons [11, 12, 15, 19, 28, 31]. MR’s immersive environment seems to provide an additional layer of cognitive support for early-career users. Interestingly, this benefit may be linked to a reduction in mental workload, as previously demonstrated by Lu et al., who used NASA-TLX metrics to assess MR-supported planning. Surgeons reported improved communication, spatial clarity, and lower frustration during decision-making when using MR environments [20]. These cognitive advantages may explain the particularly strong performance improvements in positioning and access planning observed in our junior subgroup.

Senior surgeons benefit less markedly, likely due to their pre-existing spatial visualization and procedural planning skills. However, the high level of agreement and subjective utility reported by experienced raters suggests that MR may still serve as a valuable validation tool, even for experts.

To date, few comparative studies have evaluated 3D printing and MR specifically in the context of preoperative planning for tibial plateau fractures [18, 19, 32]. While both technologies rely on similar upstream processes—namely high-resolution CT imaging and standardized segmentation—their downstream implementation differs considerably.

Once segmentation is complete, MR models can be rendered and reviewed almost immediately, whereas 3D printing typically requires additional production time, depending on printer type and materials. Depending on fracture complexity, segmentation and preparation of fracture models for MR visualization may require approximately 1–2 h per case. In contrast, preparation for 3D printing often requires additional manual post-processing steps, including removal or stabilization of floating fracture fragments and creation of watertight model surfaces to reduce printing errors and material intrusion into the model. These additional preparation steps may require approximately 0.5–1 h of further processing time.

Consequently, implementation of MR-based workflows still requires substantial technical infrastructure and expertise. Reliable MR visualization depends on standardized imaging protocols, dedicated segmentation software, and users experienced in medical image processing. While commercially available MR platforms may provide relatively streamlined visualization workflows, open-source solutions often require considerably greater manual configuration and technical expertise, particularly when integrating custom visualization environments.

In addition, reproducibility across institutions may currently be influenced by differences in imaging protocols, software environments, hardware availability, and local technical expertise. While commercial software solutions may provide relatively standardized workflows, their implementation is associated with substantial financial costs. In contrast, open-source platforms may offer greater accessibility but often require increased workflow customization, which may affect reproducibility.

Practical barriers to broader clinical implementation include integration into highly restricted institutional IT infrastructures, data protection considerations related to patient imaging data, hygiene requirements for intraoperative use, acquisition and licensing costs, and the personnel effort associated with model preparation and workflow integration.

MR environments such as those deployed with HoloLens 2 offer the theoretical advantage of hands-free interaction and wearable visualization, which may facilitate use in sterile environments. However, practical implementation in the operating room remains challenging and is currently limited by ergonomic constraints and workflow integration [33].

The use of head-mounted displays in a sterile operating room environment raises several ergonomic and workflow-related challenges, including limited software integration [34], potential discomfort during prolonged use [35], and restricted field of view, which may lead to visual distraction or reduced situational awareness [36]. In addition, user-related factors such as dizziness, eye strain, cognitive load, and fatigue have been reported and may influence usability and clinical acceptance, particularly during longer procedures [33, 35]. Furthermore, current MR systems remain limited by hardware constraints, including device bulkiness, tracking accuracy, and integration into existing surgical workflows, which may complicate intraoperative implementation [33, 37].

In contrast, 3D printing raises regulatory concerns regarding sterilization and intraoperative use, particularly when intended for direct application in the surgical field [38].

Therefore, while MR demonstrates clear potential as a preoperative cognitive support tool, further studies are required to evaluate its feasibility, safety, and added value in intraoperative settings.

### Limitations

Our study has several limitations. First, the CT scans were acquired at multiple institutions, and although all included datasets met the minimum resolution threshold for segmentation, variations in scan quality and protocol may have introduced heterogeneity in 3D model fidelity. However, to ensure consistency in model geometry, all DICOM datasets were processed using a standardised segmentation protocol and STL export workflow.

Second, participant fatigue may have influenced scoring accuracy, as the rating process involved detailed review of 22 cases across four modalities. Although breaks were permitted and the sequence of cases randomized, cognitive fatigue cannot be fully excluded.

Third, no formal training module was implemented prior to MR assessment. While participants were given brief instruction, variability in MR familiarity may have influenced performance—particularly among less experienced raters. Standardized MR training protocols may further improve reproducibility and interobserver agreement by reducing variability in user familiarity and interaction with MR environments.

In addition, the sequential presentation of visualization modalities may have introduced a carryover or learning effect, as fracture understanding could progressively increase with each subsequent assessment step. Consequently, MR—being consistently presented as the final modality—may have benefited from cumulative fracture comprehension acquired through prior evaluation using CT, 3DCT, and 3D-printed models. To partially reduce potential ordering effects, fracture cases were presented in randomized order across raters.

Fourth, this was an interobserver reliability study and did not assess intrarater reproducibility or direct clinical outcomes such as intraoperative times, quality of reduction, or complication rates. However, the inclusion of a large and heterogeneous group of raters across all experience levels, combined with a standardized and blinded assessment protocol, strengthens the generalizability and robustness of the observed agreement patterns.

Fifth, the relatively small number of included cases represents an additional limitation of this study. Although consistent trends were observed across modalities, the limited sample size may have affected the stability of agreement measures and contributed to the generally low kappa values observed across all visualization techniques. However, comparable sample sizes are frequently reported in interobserver studies investigating fracture classification and preoperative planning, primarily due to the substantial effort required for case preparation, segmentation, and standardized evaluation across multiple modalities [17, 19].

In addition, heterogeneity in fracture morphology, particularly in complex AO/OTA type C fractures, may have further influenced interobserver variability. Therefore, the generalizability of the findings remains limited and should be interpreted with caution.

Finally, variations in the technical capabilities of extended reality platforms (e.g., field of view, model resolution) complicate direct comparisons across studies. Standardization in MR model preparation and rendering remains an important prerequisite for multicenter validation. Despite these limitations, the use of high-quality segmentation protocols, multiple raters of varying experience, and comparison across four modalities provides a robust foundation for interpreting the observed improvements in agreement and planning confidence.

### Data protection and regulatory considerations

In addition to methodological limitations, data protection and regulatory aspects must be considered when implementing MR-based visualization workflows in clinical practice. All DICOM datasets were fully anonymised prior to processing, ensuring that no patient-identifiable information was included in the generated 3D models.

Segmentation and STL generation were performed locally within the institutional infrastructure using dedicated clinical workstations. The questionnaire was conducted via a GDPR-compliant survey platform with servers located in Germany (SoSci Survey GmbH).

MR visualisation was performed using a cloud-based application; however, only anonymised 3D models were used, preventing any direct linkage to patient identity. Nevertheless, the use of cloud-based environments highlights the importance of strict data governance, institutional oversight, and regulatory compliance when integrating MR technologies into clinical workflows. Future technical developments may enable fully local processing pipelines, further enhancing data security and facilitating broader clinical implementation.

### Outlook

Future research should focus on the technical and clinical standardization of MR-assisted planning in orthopaedic trauma care. To enable comparability across studies, consensus is needed on hardware specifications, file formats, rendering quality, and segmentation protocols for extended reality applications. Comparative studies that benchmark various MR platforms and software environments will be essential to define technical benchmarks and usability standards. Furthermore, it is essential to define and investigate meaningful clinical outcome parameters that are currently lacking in most MR studies. Multicenter, prospective trials should integrate MR with intraoperative navigation systems to evaluate workflow compatibility and to explore potential synergy between technologies. Larger case cohorts will be required to enable more robust statistical analyses and to validate the observed agreement patterns.

## Conclusion

Mixed Reality (MR) enhances interobserver agreement in key domains of preoperative planning for tibial plateau fractures, particularly in surgical approach selection and patient positioning. These benefits were most notable among less experienced surgeons, underscoring the value of MR as a cognitive support tool. Compared to conventional imaging and 3D printing, MR offers dynamic interaction, greater flexibility, and workflow efficiency. While further clinical studies are required to validate its intraoperative and patient-centered benefits, our findings support the integration of MR into modern orthopaedic trauma planning as a valuable adjunct across experience levels.

## Electronic Supplementary Material

Below is the link to the electronic supplementary material.

Supplementary Material 1

## Data Availability

The datasets generated and/or analysed during the current study are not publicly available due to data protection and ethical restrictions but are available from the corresponding author on reasonable request and subject to institutional approval.
