# Supplementary material for: Mixed reality improves agreement on surgical approach selection and patient positioning in tibial plateau fracture planning compared to CT, 3DCT and 3D printing
Source: Eur J Trauma Emerg Surg. 2026 Jun 22;52(1):204. doi: 10.1007/s00068-026-03230-4 (PMC13287219; doi:10.1007/s00068-026-03230-4)
Supplement: Supplementary file 1 — Supplementary Material 1 [file 68_2026_3230_MOESM1_ESM.docx]

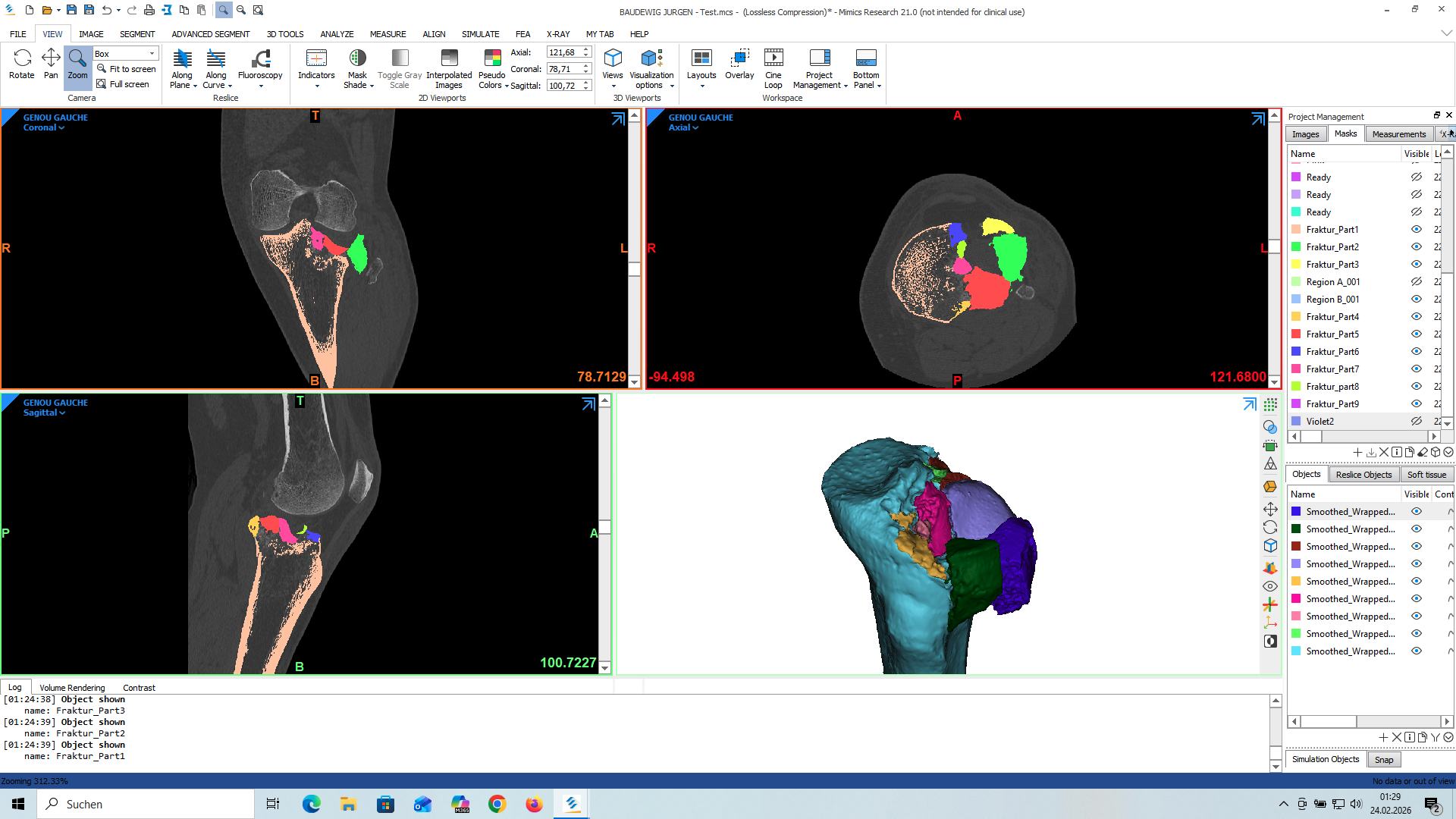


Supplement 1: threshold-based semi-automated segmentation using Materialise’s Interactive Medical Image Control System (Mimics Innovation Suite v24; Materialise, Leuven, Belgium)


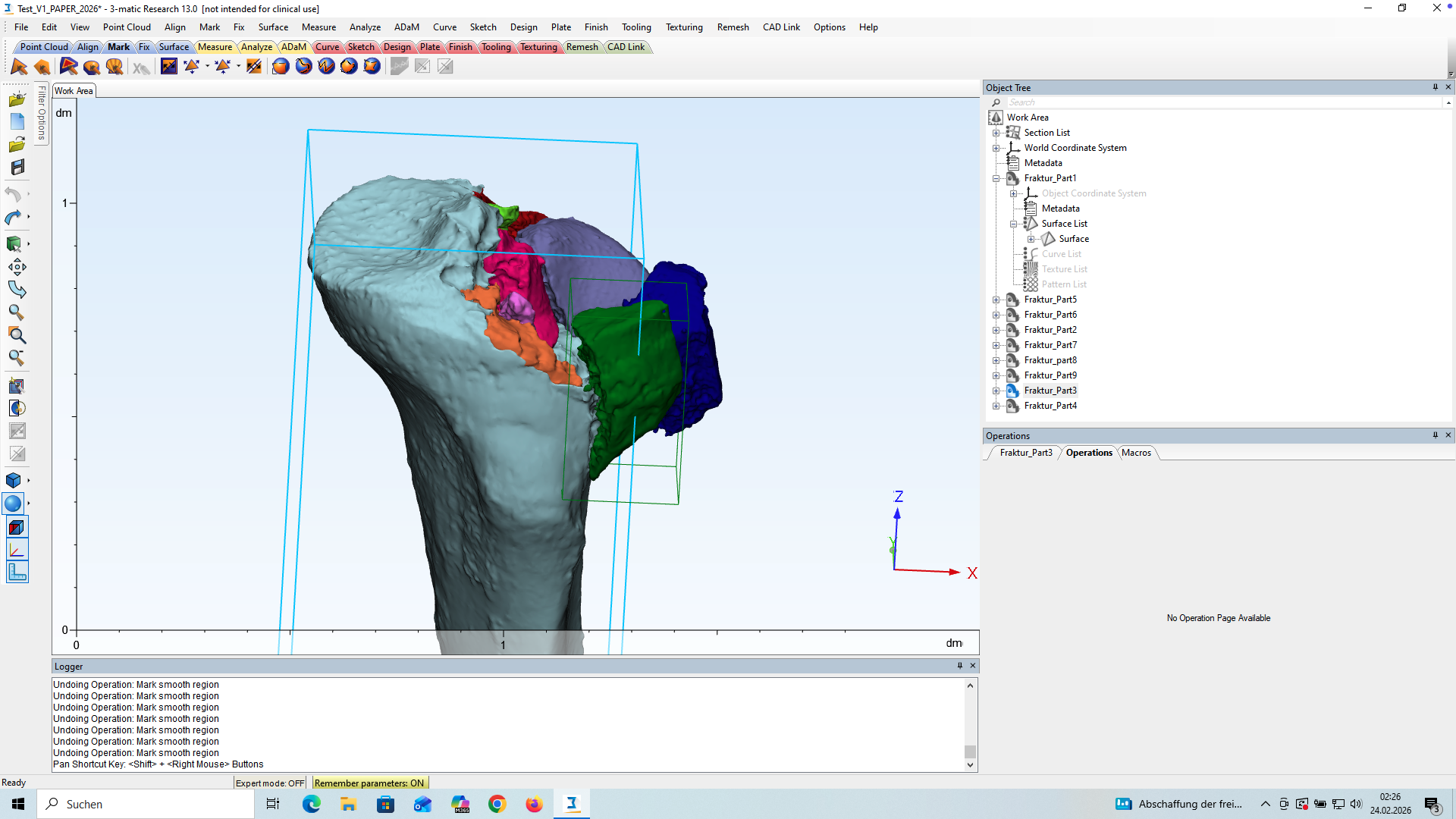

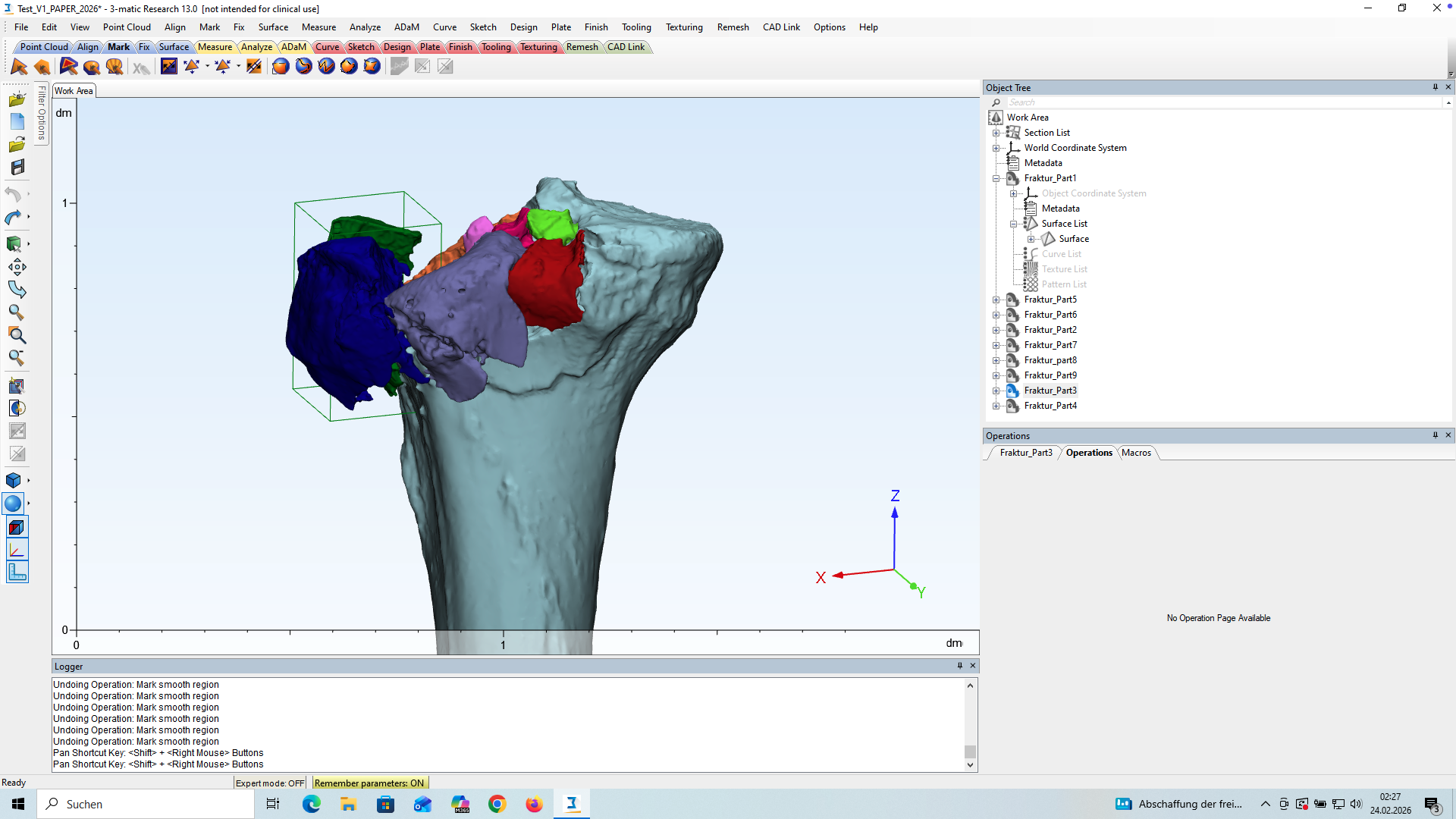

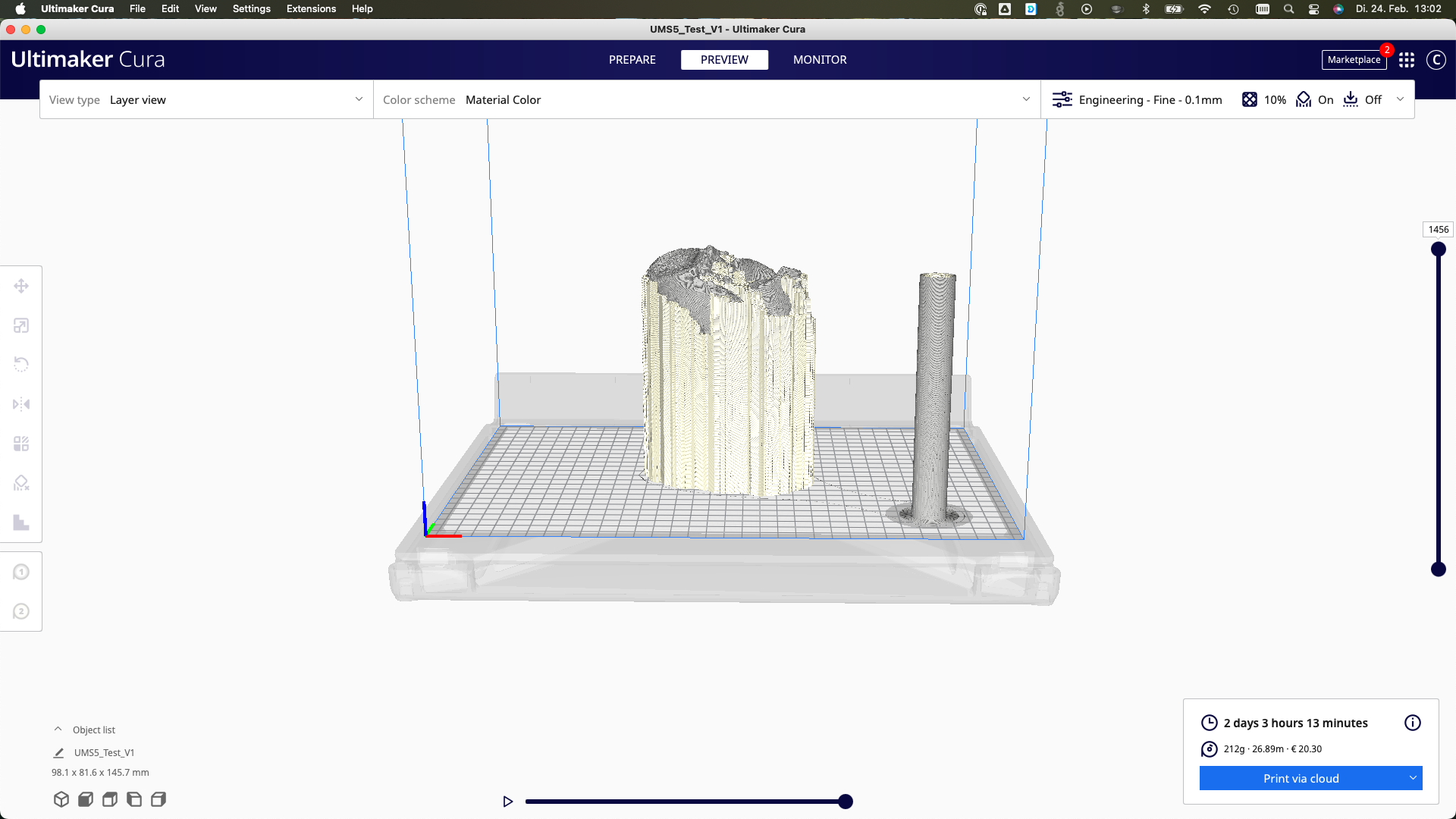

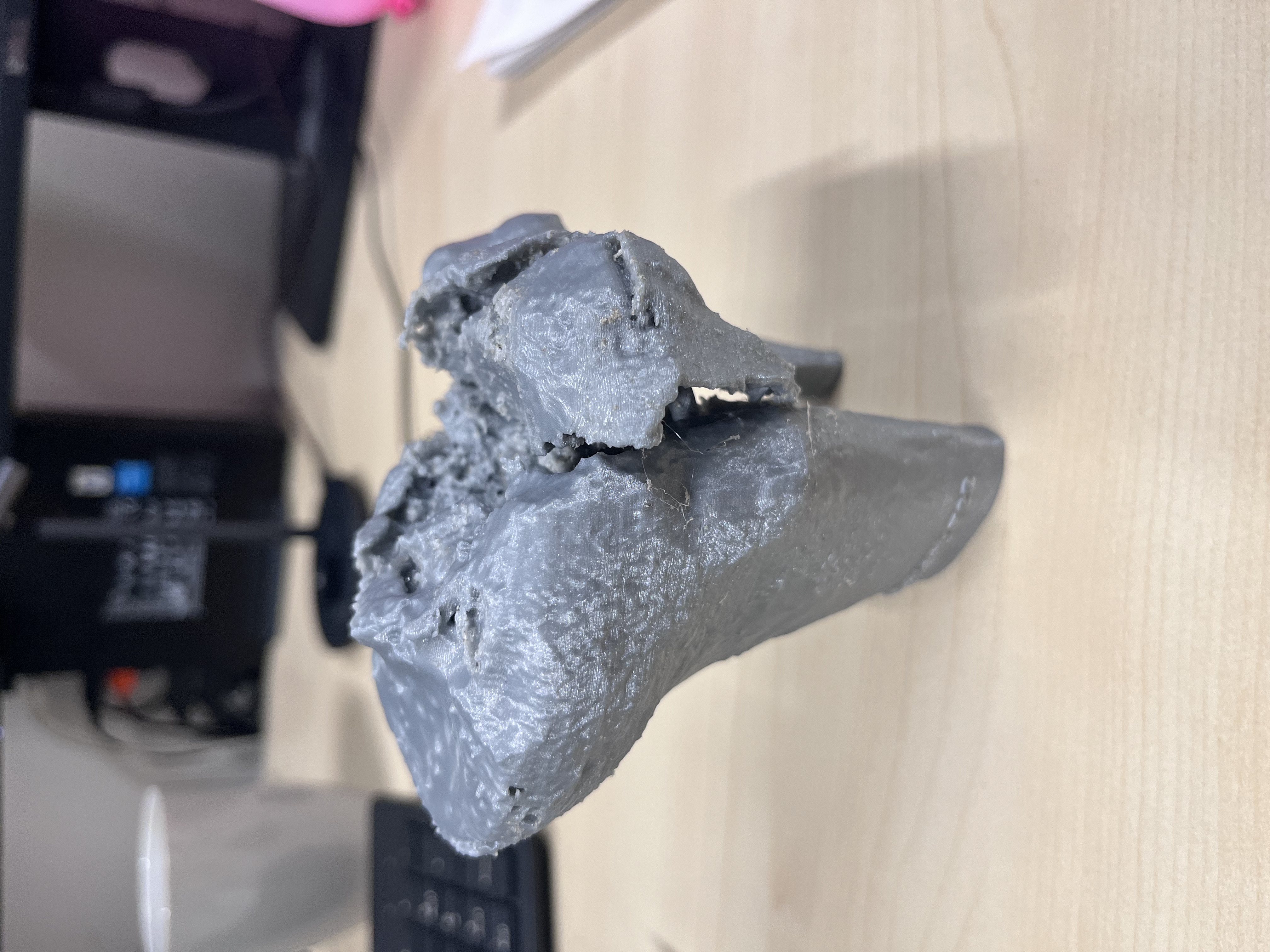


A

D

B

C

Supplement 2: post processing using Materialise 3-Matic (Materialise 3-Matic Medical v16; Materialise, Leuven, Belgium) (C), slicing process using Cura (Ultimaker Cura v4.10; Ultimaker, Utrecht, Netherlands) (D)

Supplement 3: Mixed reality setup for fracture evaulation. Study participant using the HoloLens 2 headset during case evaluation (A). Holographic visualization of a multifragmentary tibial plateau fracture from the user’s perspective, with individually color-coded fragments displayed in the Materialise Viewer application (B, C).

Supplement 4: Digital questionnaire interface used for the study. Case summary screen enabling free-text input and clinical annotations (A) and structured input section for selecting treatment concept and patient positioning (B).

| *treatment concept* | *options* |
| --- | --- |
| Treatment concept | conservative  open surgical  (only) arthroscopy  fracturoscopy |
| Patient positioning | prone positioning  supine positioning  lateral positioning  floating positioning  supine positioning with intraoperative repositioning in prone position  supine positioning with intraoperative repositioning in lateral position  prone positioning with intraoperative repositioning in supine position  prone positioning with intraoperative repositioning in lateral position  lateral positioning with intraoperative repositioning in prone position  lateral positioning with intraoperative repositioning in supine position |
| Surgical approach | anterior-lateral approach  anterior-medial approach  posterior-medial approach  posterior-lateral approach  extended medial approach  extended lateral approach  posterior approach |
| choice of implants | 3.5 mm lateral proximal tibia  3.5 mm medial proximal tibia  3.5 mm posterolateral proximal tibia  3.5 mm posteromedial proximal tibia  *Smith&Nephew, EVOS small fragment, proximal tibia steel plates* |

Supplement 5: the different aspects and their options to choose from of the treatment concept

| *κ co-efficient* | *reliability grading* |
| --- | --- |
| < 0.00 | poor |
| 0.01 – 0.20 | slight |
| 0.21 – 0.40 | fair |
| 0.41 – 0.60 | moderate |
| 0.61 – 0.80 | substantial |
| >0.80 | excellent |

Table 1: Landis and Koch grading of reliability based on κ co-efficient values^39^

| *Surgical approach* | *CT* | | *3DCT* | | *3D* | | *MR* | |
| --- | --- | --- | --- | --- | --- | --- | --- | --- |
|  | *PM* | *κ* | *PM* | *κ* | *PM* | *κ* | *PM* | *κ* |
| Overall | 29% | 0.23 | 29% | 0.22 | 33% | 0.27 | 32% | 0.30 |
| Junior Surgeons | 27% | 0.19 | 27% | 0.19 | 34% | 0.26 | 39% | 0.28 |
| Senior Surgeons | 33% | 0.27 | 33% | 0.26 | 32% | 0.26 | 31% | 0.21 |

Table 2: interobserver agreements for the surgical approach

| *Patient positioning* | *CT* | | *3DCT* | | *3D* | | *MR* | |
| --- | --- | --- | --- | --- | --- | --- | --- | --- |
|  | *PM* | *κ* | *PM* | *κ* | *PM* | *κ* | *PM* | *κ* |
| Overall | 46% | 0.25 | 46% | 0.26 | 53% | 0.36 | 57% | 0.35 |
| Junior Surgeons | 42% | 0.19 | 43% | 0.23 | 52% | 0.35 | 57% | 0.39 |
| Senior Surgeons | 55% | 0.36 | 53% | 0.33 | 55% | 0.36 | 55% | 0.27 |

Table 3: interobserver agreements for the patient positioning

| *Treatment concept* | *CT* | | *3DCT* | | *3D* | | *MR* | |
| --- | --- | --- | --- | --- | --- | --- | --- | --- |
|  | *PM* | *κ* | *PM* | *κ* | *PM* | *κ* | *PM* | *κ* |
| Overall | 83% | 0.23 | 83% | 0.25 | 82% | 0.17 | 91% | 0.11 |
| Junior Surgeons | 82% | 0.20 | 82% | 0.21 | 81% | 0.16 | 91% | -0.3 |
| Senior Surgeons | 83% | 0.24 | 84% | 0.25 | 83% | 0.15 | 92% | 0.23 |

Table 4: interobserver agreements for the treatment concept
